# MetaMSD: meta analysis for mass spectrometry data

**DOI:** 10.7717/peerj.6699

**Published:** 2019-04-10

**Authors:** So Young Ryu, George A. Wendt

**Affiliations:** 1 School of Community Health Sciences, University of Nevada - Reno, Reno, NV, United States of America; 2 Department of Epidemiology, University of California, Berkeley, Berkeley, CA, United States of America

**Keywords:** Mass Spectrometry, Differential Proteins, Proteomics, Meta-Analysis

## Abstract

Mass spectrometry-based proteomics facilitate disease understanding by providing protein abundance information about disease progression. For the same type of disease studies, multiple mass spectrometry datasets may be generated. Integrating multiple mass spectrometry datasets can provide valuable information that a single dataset analysis cannot provide. In this article, we introduce a meta-analysis software, MetaMSD (Meta Analysis for Mass Spectrometry Data) that is specifically designed for mass spectrometry data. Using Stouffer’s or Pearson’s test, MetaMSD detects significantly more differential proteins than the analysis based on the single best experiment. We demonstrate the performance of MetaMSD using simulated data, urinary proteomic data of kidney transplant patients, and breast cancer proteomic data. Noting the common practice of performing a pilot study prior to a main study, this software will help proteomics researchers fully utilize the benefit of multiple studies (or datasets), thus optimizing biomarker discovery. MetaMSD is a command line tool that automatically outputs various graphs and differential proteins with confidence scores. It is implemented in R and is freely available for public use at https://github.com/soyoungryu/MetaMSD. The user manual and data are available at the site. The user manual is written in such a way that scientists who are not familiar with R software can use MetaMSD.

## Introduction

Mass spectrometry can identify and quantify thousands of proteins simultaneously in complex biological samples (e.g., urine, serum). In particular, detecting changes in protein expression due to genetic or environmental perturbations of an organism is an important topic in biology and medicine. To estimate changes in protein expression, various methods such as stable isotope-labeling methods (e.g., ICAT (Gygi et al., 1999), iTRAQ (Hardt et al., 2005), SILAC (Ong et al., 2002)) and label-free methods  (Radulovic et al., 2004; Ryu et al., 2008; Ryu et al., 2014; Liu, Sadygov & Yates, 2004) have been developed. Normally, researchers are interested in identifying as many differential proteins as possible between two groups (e.g., normal vs. cancer) while controlling the number of false positives. In clinical and biological studies, researchers often conduct multiple proteomic studies by design (e.g., a pilot study prior to a main study). There is also a growing movement for researchers to share their mass spectrometry data, thus more datasets for the same type of studies will become available in public repositories (e.g., proteomeXchange, PRIDE). These experiments may be generated from various instruments in different laboratories using either labeling or label-free approaches. Thus, it is often more adequate to analyze each dataset with appropriate bioinformatic tools (i.e., MaxQuant (Cox & Mann, 2008), ASAPRatio (Li et al., 2003), QSpec (Choi, Fermin & Nesvizhskii, 2008), MSstats (Choi et al., 2014; Clough et al., 2012)) and combine them using a meta-analysis technique.

Meta-analysis techniques that integrate data or results from multiple studies have been widely used in health policy, medicine, and genomics  (Hedges & Olkin, 1985; Stangl & Berry, 2000). Here, we introduce MetaMSD that analyzes multiple proteomics datasets that may be generated by different labeling techniques and/or different types of mass spectrometry instruments. MetaMSD uses statistical summary combination approaches rather than normalizing expression profiles across different experiments since peptide expression profiles can be very different across multiple studies (or datasets) (Ryu et al., 2014). Noting that currently, many proteomics researchers do not utilize datasets generated for their pilot studies, we anticipate that this software can benefit proteomics researchers who want to use a meta-analysis technique to maximize their biomarker discovery. In this paper, we introduce meta-analysis techniques of MetaMSD and demonstrate the performance of the MetaMSD using both simulated and real mass spectrometry datasets. We then discuss limitations and potentials of MetaMSD.

## Materials and Methods

### MetaMSD

#### Meta-analysis methods

**Figure 1:**
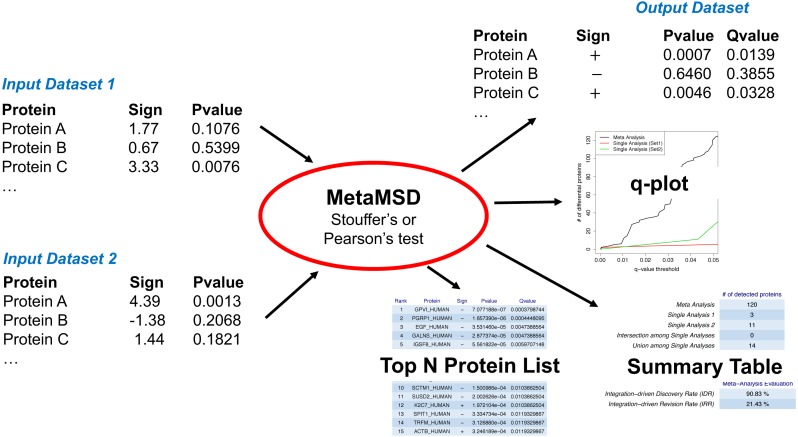
A workflow of MetaMSD. MetaMSD takes multiple quantitative proteomics results as input and generates various outputs such as meta-analysis *p*-values & q-values. It also generates graphs and tables that describe various aspects of differential proteins (e.g., q-plot, meta-analysis diagnosis).

MetaMSD combined differential protein results of multiple datasets (e.g., datasets from pilot and main studies) using Pearson’s test or Stouffer’s test (Fig. 1). Both tests were well-known meta-analysis tests that considered the directionality of hypotheses. Thus, it objectively resolved contradicting results between datasets. For example, a protein abundance of interest may be significantly larger in Group 1 than 2 for one dataset, but significantly smaller in Group 1 than 2 for another dataset. Based on one-sided *p*-value information, Pearson’s and Stouffer’s tests determined whether a protein of interest was more abundant in Group 1 or 2. Moreover, the resulting *p*-values/*q*-values reflected uncertainty of this decision. It was previously known that Pearson’s method was more sensitive to large *p*-values than Stouffer’s method, while Stouffer’s method was more sensitive to small *p*-values than Pearson’s method (Heard & Rubin-Delanchy, 2018). Researchers may choose one of these two meta-analyses approaches for their experiments depending on their study goals. Here, we elaborated both methods in terms of proteins and datasets. We let ${p}_{i,j}^{L}$ be a *p*-value of the one-sided (left) hypothesis test for *j*th protein using the *i*th dataset (study) where *i* = 1, …, *K* and *j* = 1, …, *N*. The number of hypothesis tests (or proteins) was denoted as *N* and the number of datasets (or studies) was denoted as $K\cdot ~{p}_{i,j}^{L}$ was a probability of falsely concluding that the *j*th protein in the *i*th dataset was less abundant in Group 1 than Group 2. Thus, as ${p}_{i,j}^{L}$ became smaller, we were more confident that the *j*th protein in the *i*th dataset was less abundant in Group 1 than Group 2. Similarly, if a right-sided *p*-value, ${p}_{i,j}^{R}$, was less than 0.05, then we concluded that the mean abundance of the *j*th protein in the *i*th study was significantly higher in Group 1 compared to Group 2 at the 95% confidence level. In mathematical notation, ${p}_{i,j}^{R}=1-{p}_{i,j}^{L}$. Thus, as ${p}_{i,j}^{L}$ became larger, ${p}_{i,j}^{R}$ became smaller.

Pearson’s test was a one-sided Fisher’s test. It took a minimum *p*-value of left- and right-sided Fisher’s tests with a Bonferroni correction. Different from Fisher’s method, it considered the directionality of the hypotheses. Specifically, Pearson’s test was based on the following procedures:

(1) \begin{eqnarray*}{Q}_{j}^{T}=max({Q}_{j}^{L},{Q}_{j}^{R}),\end{eqnarray*}

where ${Q}_{j}^{L}=-2log({\mathop{\prod }\nolimits }_{i=1}^{K}{p}_{i,j}^{L})$ and ${Q}_{j}^{R}=-2log({\mathop{\prod }\nolimits }_{i=1}^{K}(1-{p}_{i,j}^{L}))$. And a *p*-value based for the *j*th protein was ${p}_{j}=min(1,2Pr({\chi }_{2K}^{2}\geq {Q}^{T}))$. Stouffer’s test used the sum of inverse normal transformation of *p*-values. Stouffer’s statistics was:

(2) \begin{eqnarray*}{Z}_{j}= \frac{\sum _{i=1}^{K}{Z}_{i,j}}{\sqrt{K}} \end{eqnarray*}

where *Z*_*i*,*j*_ was converted from a *p*-value from the *i*th dataset (or study) and the *j*th protein. Then, its left-sided *p*-value for the *j*th protein, ${p}_{j}^{L}$, was calculated based on *Z*_*j*_ which had a standard normal distribution under the null hypothesis. Its right-sided *p*-value was ${p}_{j}^{R}=1-{p}_{j}^{L}$. The final *p*-value was calculated by taking a side with a smaller one-sided *p*-value and applying a Bonferroni correction. For both Pearson’s and Stouffer’s tests, we considered all the proteins that were quantified in at least one of the studies, thus *K* may vary from one protein to another. If a protein was quantified in more than one study, then the meta-analysis was employed. If a protein was quantified in only one study, then the *p*-value from that one study was kept without employing the meta-analysis. Since we usually performed many hypothesis tests (e.g., 6,000 to 10,000 tests), we employed a false discovery procedure to correct multiple testing errors. MetaMSD reported *q*-values, which were the minimum false discovery rates that could be attained when calling that the abundances of proteins were significantly different between groups (Storey, 2002).

MetaMSD also measured a meta-analysis performance using the Integration-driven Discovery Rate (IDR) and the Integration-driven Revision Rate (IRR). Average IDR and IRR were reported for the simulation study. IDR represented a proportion of proteins detected by the meta-analysis that were not discovered in any of the individual studies. IRR represented a proportion of proteins detected in at least one individual study, but not by the meta-analysis. Thus, we wanted our meta-analysis method to give a higher IDR without unnecessarily increasing IRR at a given false discovery rate threshold.

#### MetaMSD software

MetaMSD took signs of test statistics and the corresponding *p*-values as input and generated meta-analysis *p*-values, the directionality of hypothesis tests, and the corresponding *q*-values as outputs (Fig. 1). Noting that major proteomic quantification software can generate test statistics and *p*-values, MetaMSD can be used to integrate protein quantification results generated from various proteomic software.

**Figure 2:**
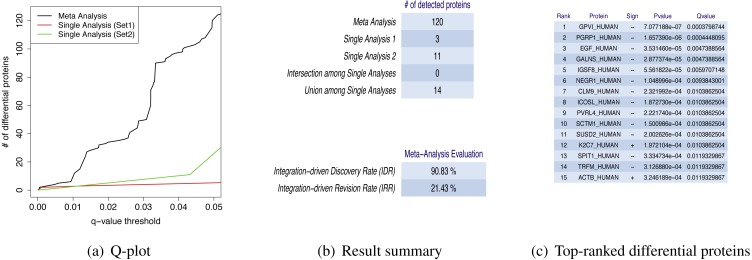
MetaMSD sample outputs. (A) The *q*-value threshold vs. # of differential proteins detected by meta-analysis (Stouffer’s test) and single dataset analyses. (B) The numbers of detected proteins and meta-analysis evaluation measures. The top table contains results for a meta-analysis (Stouffer’s test), single analyses using Dataset 1 and 2, the number of commonly detected proteins between single analyses (intersection among Single Analyses), and the number of any proteins detected by single analyses (union among Single Analyses). The bottom table shows the performance measures of the meta-analysis using IDD (Integration-driven Discovery Rate) and IDR (Integration-driven Revision Rate). (C) Top-N differential proteins list ranked by their *p*-values. MetaMSD displays top-ranked protein names, their signs of log-transformed difference between comparison groups, *p*-values and *q*-values.

MetaMSD generated a tab-delimited file that contained both single dataset analyses results and a meta-analysis result. The file contained protein name, the directionalities of hypothesis tests, *p*-values and *q*-values for single analyses and meta-analysis results. It also generated several graphs/tables that described various aspects of differential proteins: (1) a plot that described numbers of detected differential proteins given *q*-value thresholds (Fig. 2A); (2) Summary statistics about the numbers of detected proteins and a meta-analysis diagnosis (Fig. 2B); and (3) Top-N differential proteins list detected by meta-analysis (Fig. 2C). The following command line generated a result file and graphs/tables:

**Table:** 

*MetaMSD.Rscript [options]*
Available options were:
–*metaanalysis=Stouffer*
Specify a meta-analysis test. User can choose either Stouffer or Pearson (default=Stouffer).
*–cutoff=0.05*
Specify a *q*-value cut off (default=0.05).
*–top=15*
Specify the number of proteins in Top-N differential protein list (default=15).
*–input=input*
Specify the input folder name (default=input).
*–output=output*
Specify the output folder (default=output).
*–help*
Show help message and exit.

The software MetaMSD, its manual, and example datasets are available at https://github.com/soyoungryu/MetaMSD under the terms of the MIT license, which allows further community driven meta-analysis development.

### Data

#### Simulated datasets

MetaMSD was tested using ten different simulation scenarios. For each simulation scenario, we generated one thousand simulations and calculated average performance measures of meta-analysis methods. For each simulation, multiple datasets (or studies) were generated and analyzed using MetaMSD. Each dataset contained 2*n* samples with *n* samples per group. A total of 2,000 proteins were generated per study. We varied the number of samples per group, *n*, where *n* =6, 9, or 12. Mass spectrometry was often not able to quantify all proteins in complex mixtures, and a protein quantified in one dataset might not be necessarily quantified in another dataset. Thus, we let the overlap percentage in quantified proteins between datasets to be 75% (*ρ* = 0.75) for the most of simulations. However, different *ρ* values were also explored and discussed. The data were simulated using the following schemes:

(3) \begin{eqnarray*}{x}_{ij}& \sim N({\gamma }_{j},{\lambda }_{j}^{2}),\end{eqnarray*}

(4) \begin{eqnarray*}\log \nolimits ({\gamma }_{j})& \sim N(\mu ,{\sigma }^{2}),\end{eqnarray*}

(5) \begin{eqnarray*}\log \nolimits ({\lambda }_{j})& \sim N(\alpha +\beta log({\gamma }_{j}),\eta ),\end{eqnarray*}

where *x*_*ij*_ was an abundance for protein *j* in dataset *i*, *γ*_*j*_ was a mean abundance for protein *j*, and *λ*_*j*_ was its standard deviation. In Eq. (5), we specified *λ*_*j*_ such that there was a linear relationship between *log*(*λ*_*j*_) and *log*(*γ*_*j*_). In other words, more abundant proteins had higher variance. The parameters were set as the followings: *μ* = 2.42, *σ* = 0.30, *α* = 1.63, *β* =  − 0.90, and *η* = 0.50. These values were obtained from label-free plasma proteomic data of mice (Kuusela et al., 2017). Raw data are available via ProteomeXchange with identifier PXD005022. The last three parameters were estimated by fitting a linear regression on log-transformed means of protein abundances and the corresponding log-transformed standard deviations. The simulated data and mouse data had similar protein abundance distributions including the mean–variance relationship. In our simulated dataset, 30% of proteins were differential proteins with 1.5, 2, or 4 fold changes in protein abundances between experiments. *T*-tests (with unequal variances) were performed for each dataset, then meta-analysis techniques were applied to combine resulting *p*-values. The following was a summary of 10 simulation scenarios:

Simulation Scenario 1: ***n*** = **6**, *ρ* = 0.75, *K* = 2, *α* = 1.63

Simulation Scenario 2: ***n*** = **9**, *ρ* = 0.75, *K* = 2, *α* = 1.63

Simulation Scenario 3: ***n*** = **12**, *ρ* = 0.75, *K* = 2, *α* = 1.63

Simulation Scenario 4: *n* = 6, *ρ* = 0.75, ***K*** = **3**, *α* = 1.63

Simulation Scenario 5: *n* = 6, *ρ* = 0.75, ***K*** = **4**, *α* = 1.63

Simulation Scenario 6: *n* = 6, *ρ* = 0.75, ***K*** = **5**, *α* = 1.63

Simulation Scenario 7: *n* = 6, *ρ* = 0.75, *K* = 2, *α* = 1.63 × 1.5

Simulation Scenario 8: *n* = 6, *ρ* = 0.75, *K* = 2, *α* = 1.63 × 2.0

Simulation Scenario 9: *n* = 6, *ρ* = 0.50, *K* = 2, *α* = 1.63

Simulation Scenario 10: *n* = 6, *ρ* = 0.25, *K* = 2, *α* = 1.63

Simulation Scenario 1 was used as a baseline scenario. Using Simulation Scenarios 1–3, we investigated MetaMSD performance for varying sample size (*n* = 6, 9, or 12). Based on Simulation Scenarios 1 and 4–6, we investigated MetaMSD performance for varying number of studies. Simulation Scenarios 1 and 7–8 were used to investigate the effects of protein expression level variability and varying experiment qualities on the meta-analysis results. Using Simulation Scenarios 1, 9 and 10, we observed how different overlap percentages in quantified proteins between studies affected meta-analysis results.

#### Kidney transplant proteomic datasets

Two urinary proteomic datasets of renal transplant patients were obtained from ProteomeXchange (Vizcaino et al., 2014) (PXD 002761). The details about mass spectrometry experiments can be found in Sigdel et al. (2014); Sigdel et al. (2016). In brief, these datasets contained four phenotypes of kidney transplant patients. However, in this paper, the focus was on a two-group comparison by comparing acute allograft rejection (AR) and stable allograft (STA) of kidney transplant patients. The first dataset was from an iTRAQ (Hardt et al., 2005) mass spectrometry study. Each pooled sample generated from either five patients with acute rejection (AR) or five patients with stable graft function (STA). The data were analyzed by DeconMSn (Mayampurath et al., 2008) and Sequest (Eng, McCormack & Yates, 1994). The *p*-values were obtained by comparing six pooled AR samples and six pooled STA samples using *t*-tests with unequal variances. The second dataset was from a label-free mass spectrometry study. There were 40 AR samples and 40 STA samples. However, to demonstrate the usefulness of the meta-analysis approaches in combining small- to large-scale studies, we varied the sample size for AR patients and for STA patients with *n*_*A*_ =6, 9, 12, or 40. The label-free dataset was analyzed by MaxQuant (Cox & Mann, 2008) and MSstats (Choi et al., 2014; Clough et al., 2012). We combined the *p*-values generated from the iTRAQ and label-free studies using MetaMSD.

#### Breast cancer proteomic datasets

Two proteomic datasets of estrogen receptor-positive (ER^+^) and triple-negative breast cancer (TNBC) breast cancer tumor samples were obtained from the following publications: Cancer Genome Atlas Network (2012) and Gámez-Pozo et al. (2015). To demonstrate the usefulness of MetaMSD in integrating medium-size datasets, we did not utilize all the patient data in these large-scale studies. Furthermore, in order to match patient’s clinical characteristics between datasets, we used the proteomic data of patients who met the following criteria: HER2-negative (Human Epidermal growth factor Receptor 2 negative) breast cancer, and Lymph node-positive with lymph node status of either N1 or N2. The first dataset (denoted as Dataset A) contained iTRAQ labeling proteomic data of 21 ER+ patients and six TNBC patients. The samples were analyzed using a nanoLC system coupled to a Q Exactive MS (Thermo Scientific, Waltham, MA, USA). The second dataset (denoted as Dataset B) contained label-free proteomic data of nine ER+ patients and nine TNBC patients. These were analyzed by a LTQ-Orbitrap Velos hybrid mass spectrometer coupled to NanoLC-Ultra system. These two clinical proteomic datasets were freely available with some restrictions. For the first dataset, protein quantification information we used in this paper, raw data, and the detailed description are freely available to the public at https://cptac-data-portal.georgetown.edu/cptac/s/S015. However, users need to agree to their data agreement policy. For the second dataset, the protein quantification information we used in this paper and detailed description about data processing are available in Gámez-Pozo et al. (2015) as Supplementary Materials. Raw mass spectrometry data are also available at Cancer Research Online (http://cancerres.aacrjournals.org/) upon the corresponding author’s approval. For both datasets, the *p*-values were calculated using *t*-tests with unequal variances and combined using MetaMSD.

## Results

### Simulation results

MetaMSD performed better than the individual analyses, detecting more differential proteins (Table 1). At a *q*-value threshold of 5%, Stouffer’s and Pearson’s tests detected 66% and 51% more differential proteins than the best individual data analysis in Simulation Scenario 1. Stouffer’s test had a higher average true integration-driven discovery rate (tIDR) than Pearson’s test. Nearly 15% of true differential proteins detected by Stouffer’s test were never detected by any individual analysis. Both Pearson’s and Stouffer’s tests had relatively low true integration-driven revision rate (tIRR), missing only less than 3% of true differential proteins detected by the individual analyses. True FDRs for both meta- and individual-analyses were less than 5%. This implied that our *p*-value/*q*-value estimations were reasonable.

**Table 1:** The performance of MetaMSD using simulated datasets (Simulation Scenario 1). The sample size for each group was 6 (*n* = 6) and the *q*-value threshold was 5%. The average numbers of detected differential proteins, the average true false discovery rates (tFDR), the average true integration-driven discovery rates (tIDR), and the average true integration-driven revision rates (tIRR) were reported. The results were based on 1,000 simulations.

Meta analysis (MetaMSD)	No. of detected proteins	Average tFDR	Average tIDR	Average tIRR
Pearson’s test	322.10	3.45%	8.20%	2.63%
Stouffer’s test	355.05	4.70%	14.66%	1.55%

Individual analysis	No. of detected proteins	Average tFDR	Average tIDR	Average tIRR
Exp 1	213.32	4.66%	.	.
Exp 2	213.25	4.60%	.	.

**Figure 3:**
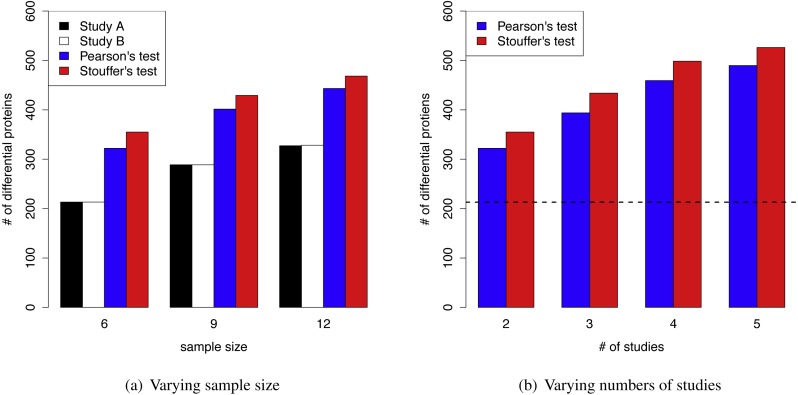
(A) MetaMSD performance for varying sample size (*n* = 6, 9, or 12) where *K* = 2 (Simulation Scenario 1, 2, and 3) (B) MetaMSD performance for varying numbers of studies (or datasets) where *K* =2, 3, 4, or 5 (Simulation Scenario 1, 4–6). The dotted horizontal line represents the number of detected proteins by an individual analysis. For both (A) & (B), the results were based on 1,000 simulations (*q* < 0.05, *ρ* = 75%). The average numbers of detected differential proteins were reported.

The superior performance of MetaMSD was observed when sample sizes were small to moderate. When the sample size was nine (*n* = 9), Stouffer’s test detected 48% more differential proteins than the individual analysis (Simulation Scenario 1 vs. 2). For a moderate sample size (*n* = 12), it detected 43% more differential proteins (Simulation Scenario 1 vs. 3). A similar pattern was observed for Pearson’s test. As a sample size increased, the number of differential proteins detected by individual analyses increased and the benefit of using meta-analyses gradually decreased (Fig. 3A).

For larger variance of protein abundances, the superior performance of MetaMSD was observed (Simulation Scenario 1, 7, and 8) as shown in Table S1. For instance, when we increased our initial *α*, thus increasing variance by 50%, 330% more differential proteins were detected by Stouffer’s tests (compared to the individual analysis). Similar results were observed for Pearson’s test with 251% improvement compared to the individual analysis. Since the simulation parameters (including the protein abundance variances) were obtained from the mouse study, we anticipated larger protein abundance variances for human disease studies. The advantages of using MetaMSD may be more pronounced for human disease studies.

Figure 3B showed that as the number of studies (or datasets) increased, meta-analyses detected more differential proteins (Simulation Scenario 1, 4–6). Stouffer’s test consistently performed better than Pearson’s test in our simulation studies in terms of the number of detected proteins.

In mass spectrometry analysis, a protein quantified in one study might not be quantified in another study. Thus, the overlap in quantified proteins between two proteomic studies would be less than perfect. To investigate its effect, we varied *ρ* to be 0.75, 0.50 and 0.25 (Simulation Scenario 1, 9–10). As the overlap of quantified proteins between studies decreased, true integration-driven discovery rates (tIDR) slightly decreased. The tIDRs for Stouffer’s test were 14.66%, 10.27%, and 5.39% when *ρ* = 0.75, 0.50 and 0.25, respectively. The average tIDRs for Pearson’s test were 8.20%, 5.26% and 2.88% when *ρ* = 0.75, 0.50 and 0.25, respectively. The average true integration revision rates were relatively consistent with less than 3% for Pearson’s test and less than 2% for Stouffer’s test (Table S2). Therefore, in order to take advantage of the meta-analysis technique, it would be important to have relatively large proportions of common quantified proteins between studies (e.g., >50% overlap).

### Application in kidney transplant proteomic data

**Figure 4:**
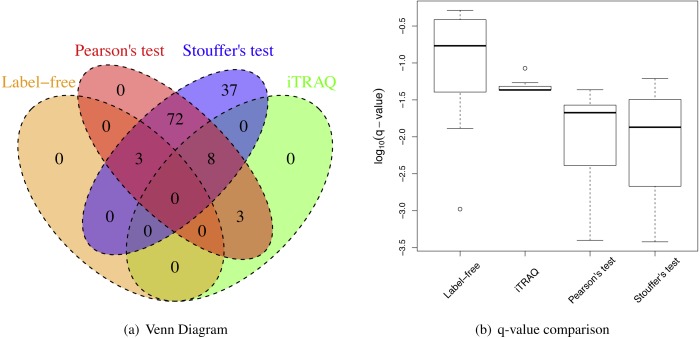
Renal transplant proteomic results. (A) A Venn Diagram for the number of differential proteins detected by individual analyses and meta-analyses. (B) The log-transformed *q*-value comparison between individual analyses and meta analyses.

**Figure 5:**
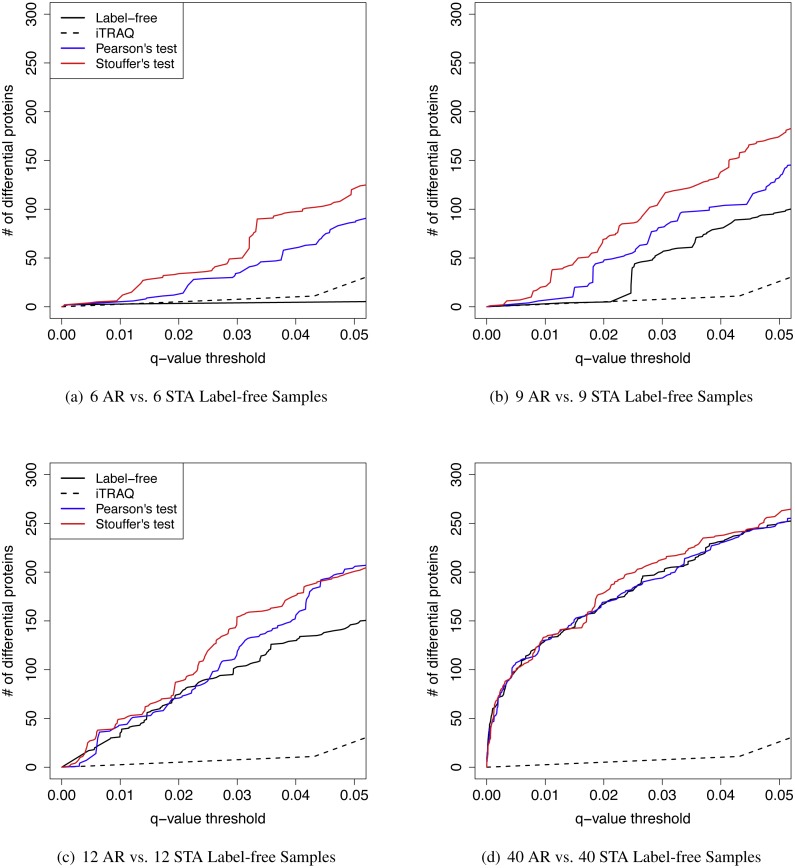
The *q*-value threshold vs. the number of differential proteins for renal transplant proteomic experiments The number of iTRAQ samples was six pooled samples per patient group. The number of label-free samples was varied. (A) Six AR vs. six STA label-free samples. (B) Nine AR vs. nine STA label-free samples. (C) 12 AR vs. 12 STA label-free samples. (D) Forty AR vs. 40 STA label-free samples.

When the sample size per group was six for both iTRAQ and label-free proteomic studies, Stouffer’s and Pearson’s test detected more differential proteins than individual analyses (Table 2). Both had very high IDRs (integration-driven discovery rate). The IDR for Pearson’s test was over 80%. The IDR for Stouffer’s test was over 90%. These implied that both Stouffer’s and Pearson’s were able to detect many differential proteins that were not detected by any individual analyses. The IRR of Pearson’s test was 0%. Thus, Pearson’s test detected all differential proteins detected by either iTRAQ or label-free studies (Fig. 4A). The IRR of Stouffer’s test was low, but higher than Pearson’s test. Stouffer’s test detected 11 out of 14 differential proteins that were detected by either iTRAQ or label-free studies. The numbers of detected proteins were 86 and 120 for Pearson’s and Stouffer’s test, respectively. MetaMSD detected 6.8 or 10.9 times more differential proteins than the individual label-free analysis. This demonstrated that researchers could detect more differential proteins at the same false discovery rates by combining available study results. Figure 4B displayed the distribution of *log*_10_ transformed *q*-values of differential proteins detected in at least one individual analysis with same consistent relative abundances in both label-free and iTRAQ analyses. These *q*-values were smaller in meta-analyses compared to individual analyses. In the renal transplant patients’ experiments, true false discovery rates were not known, thus not reported, However, *q*-values, which were minimum false discovery rates, were displayed in Fig. 5A.

**Table 2:** The number of detected proteins (compared to the best individual analysis), IDR, and IRR (FDR threshold 5%) for Renal Transplant Proteomics Data when the sample size was six per group for both iTRAQ and label-free studies.

Meta analysis	No. of detected proteins	IDR	IRR
Pearson’s test	86	83.72%	0.00%
Stouffer’s test	120	90.83%	21.43%

Individual analysis	No. of detected proteins	IDR	IRR
Label-free	3	.	.
iTRAQ	11	.	.

We further investigated the effect of various sample sizes on performance of MetaMSD (Pearson’s and Stouffer’s tests) (Fig. 5). Specifically, we varied the sample size of the label-free study as *n*_*A*_ = 6, 9, 12, or 40. We fixed the sample size of the iTRAQ study with *n*_*B*_ = 6 because only six samples per group were available for the iTRAQ study. One may view the iTRAQ study as a pilot study and the label-free study as a main study. When the main (label-free) study had a moderate sample size, Stouffer’s and Pearson’s tests consistently performed better than using only the main study result. As the sample size of the main study increased, the benefit of meta-analyses decreased. When the sample size of the main study reached 40, Stouffer’s test performed only slightly better than a main study analysis. Pearson’s test performed similarly to the main study results. This implied that when one experiment could maximize the number of differential proteins, adding an additional study did not add much value. We note that the sample size displayed in this section cannot be applicable to other studies. It may depend on the variability of samples. Our analysis was based on renal patients’ urine samples which may have relatively large biological variance.

### Application in breast cancer proteomic data

**Figure 6:**
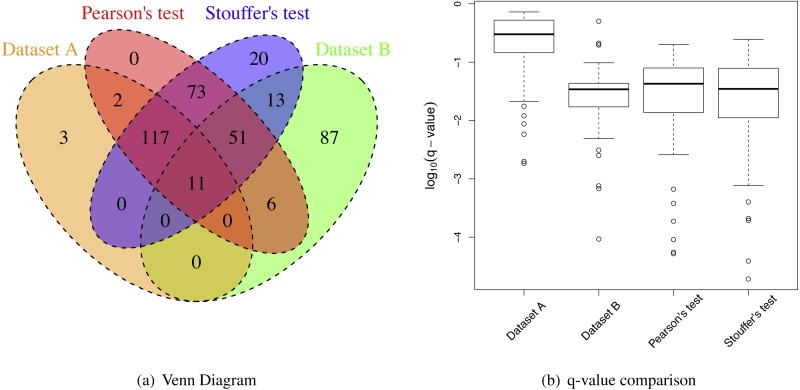
Breast cancer proteomic results. (A) A Venn Diagram for the number of differential proteins detected by individual analyses and meta-analyses. (B) The log-transformed *q*-value comparison between individual analyses and meta-analyses.

MetaMSD performed well in integrating two medium-size datasets from different laboratories (Table 3). Using Stouffer’s test, MetaMSD detected nearly 70% more differential proteins than the best individual analysis. Pearson’s test detected about 55% more differential proteins. Both meta-analysis tests had moderate IDRs with 28% for Pearson’s test and 33% for Stouffer’s test. Stouffer’s test detected 93 differential proteins that were not detected by any of individual analyses (Fig. 6A). Pearson’s test detected 73 such proteins. More than 250 differential proteins were detected by both Pearson’s and Stouffer’s tests.

**Table 3:** The number of detected proteins (compared to the best individual analysis), IDR, and IRR (FDR threshold 5%) for Breast Cancer Proteomics Data.

Meta analysis	No. of detected proteins	IDR	IRR
Pearson’s test	260	28.08%	35.52%
Stouffer’s test	285	32.63%	33.79%

Individual analysis	No. of detected proteins	IDR	IRR
Dataset A	133	.	.
Dataset B	168	.	.

However, the IRRs were more than 30% for both Pearsons’ and Stouffer’s tests, not being able to detect some proteins detected by individual analyses (Table 3 and Fig. 6A). This could be due to slightly different clinical characteristics of patients between two studies. When selecting ER^+^ and TNBC patients, we tried to match patients’ clinical characteristics between two datasets, however, there was a limitation in this approach because some clinical variables (e.g., hormonal therapy) were not available. However, some proteins had very small *q*-values after applying meta-analyses as shown in Fig. 6B. These proteins were the proteins that had small *p*-values for both studies (Dataset A and B) with the consistent relative abundances. They may be robust protein candidates for differentiating ER^+^ and TNBC patients.

## Discussion

Stouffer’s and Pearson’s tests were successful in identifying more differential proteins than an individual analysis at a given false discovery rate. We believe that our software, MetaMSD, will help scientists utilize the information from their studies at full capacity and prevent unnecessary repetition of experiments. The usage of MetaMSD is not limited to proteomics, but also to any quantitative mass spectrometry datasets including mass spectrometry-based metabolomic datasets. Thus, MetaMSD can help researchers explore publicly available mass spectrometry datasets and discover interesting biomarkers.

For the meta-analysis methods introduced in this paper, three assumptions were made. First, it was assumed that *p*-values from individual analyses were properly estimated in order to accurately control the number of false positives. Thus, if the individual analyses underestimated *p*-values, then the meta-analysis approaches that combined these *p*-values would underestimate *p*-values. In our data analysis, we made sure that we used the software/statistical approach (e.g., MSstats) that properly estimated *p*-values. The second assumption was independence of studies. In other words, we assumed that different studies did not contain measurements from the same patients (even though technical replicates were allowed within a study). For example, if Datasets X and Y contained mass spectrometry runs from the same patients, *p*-values from Dataset X and Y would be correlated. In this study, the meta-analysis methods may yield underestimated *p*-values. In our datasets, none of the patients’ urine samples were analyzed by both iTRAQ and label-free approaches to the best of our knowledge, thus *p*-values were not correlated. For breast cancer studies, patients were in different countries. However, when samples between studies are completely or partially overlapped, correlated *p*-values must be properly handled. One possible solution to handle the correlation is using a permutation approach to compute *p*-values for integrative approaches. Third, it was assumed that individual analyses were comparable to each other. As shown in breast cancer studies, more caution was needed when combining studies that were designed for different goals.

In this paper, we demonstrated the usefulness of MetaMSD in discovering more differential proteins. We note that MetaMSD is different from the approaches that combine untargeted and targeted proteomics experiments described in Li et al. (2016) and MacLean et al. (2010). In the latter approach, potential protein markers are selected in a pilot study, then re-analyzed and evaluated in a main study. Thus, in this strategy, quantification in the pilot study is not directly utilized in the final *p*-value calculation. However, MetaMSD utilizes hypothesis test results from both pilot and main studies. In addition, it is not limited to integrating pilot and main studies.

However, integrating more datasets does not always guarantee the detection of additional differential proteins. For example, if all true differential proteins were already detected at a given significance level, integrating an additional dataset will not help us detect more differential proteins except increasing our confidence about the findings. Also, the meta-analysis cannot help researchers detect extremely low abundance differential proteins that were never quantified in any of studies. However, if true differential proteins were quantified by instruments, the meta-analysis can increase our chances to detect such differential proteins by integrating more datasets. The development of a power analysis that estimates the minimum number of datasets and their sample size for the additional differential proteins detection will help researchers plan their future experiments. In the future, we plan to develop a simulation-based power analysis for MetaMSD.

In conclusion, MetaMSD is a user-friendly software that integrates multiple proteomics datasets using Stouffer’s or Pearson’s test. We believe that this software will be beneficial to scientists who cannot perform mass spectrometry analysis on a large-scale, but want to maximize their protein biomarker protein list by combining the results from their pilot and primary studies.

##  Supplemental Information

10.7717/peerj.6699/supp-1 Supplemental Information 1 Meta-analysis simulation results with different variances The average numbers of detected differential proteins were reported. The average true false discovery rates were shown in parentheses. The results were based on 1,000 simulations.

10.7717/peerj.6699/supp-2 Supplemental Information 2 Meta-analysis simulation results with different overlap percentages in quantified proteins between studies The average numbers of detected differential proteins were reported. The average true integration-drive discovery rates (tIDR) and the average true integration revision rates (tIRR) were shown in parentheses. The results were based on 1,000 simulations.

## Additional Information and Declarations

### Data Availability

The following information was supplied regarding data availability: The software MetaMSD, its manual, and example datasets are available at https://github.com/soyoungryu/MetaMSD. In this article, we used only publicly available raw proteomics datasets: (PXD 002761 and PXD005022). Breast cancer proteomics data is available at
https://cptac-data-portal.georgetown.edu/cptac/s/S015 and at the Cancer Research Online (http://cancerres.aacrjournals.org/) under breast cancer proteomics.
