# Supplementary material for: MetaMSD: meta analysis for mass spectrometry data
Source: PeerJ. 2019 Apr 10;7:e6699. doi: 10.7717/peerj.6699 (PMC6462182; doi:10.7717/peerj.6699)
Supplement: Supplemental Information 1 — The average numbers of detected differential proteins were reported. The average true false discovery rates were shown in parentheses. The results were based on 1,000 simulations. [file peerj-07-6699-s001.docx]

Supplementary Table 1. Meta-analysis simulation results with different variances (q-value threshold of 5%, n = 6, and $\rho$ = 75%). The average numbers of detected differential proteins were reported. The average true false discovery rates were shown in parentheses. The results

were based on 1,000 simulations.

| Meta Analysis | $\alpha$=1.63 | $\alpha$ =1.5*1.63 | $\alpha$ =2*1.63 |
| --- | --- | --- | --- |
| Pearson’s test | 322.10 (3.45%) | 77.99 (3.56%) | 2.17 (6.11%) |
| Stouffer’s test | 355.05 (4.70%) | 98.57 (4.30%) | 3.18 (5.97%) |
| Individual Analysis | $\alpha$ =1.63 | $\alpha$ =1.5*1.63 | $\alpha$ =2*1.63 |
| Exp 1 | 213.32 (4.66%) | 22.21 (4.51%) | 0.34 (19.52%) |
| Exp 2 | 213.24 (4.60%) | 22.21 (4.37%) | 0.33 (19.23%) |
