# Supplementary material for: MetaMSD: meta analysis for mass spectrometry data
Source: PeerJ. 2019 Apr 10;7:e6699. doi: 10.7717/peerj.6699 (PMC6462182; doi:10.7717/peerj.6699)
Supplement: Supplemental Information 2 — The average numbers of detected differential proteins were reported. The average true integration-drive discovery rates (tIDR) and the average true integration revision rates (tIRR) were shown in parentheses. The results were based on 1,000 simulations. [file peerj-07-6699-s002.docx]

Supplementary Table 2. Meta-analysis simulation results with different overlap percentages ($\rho)$ in quantified proteins between studies (q-value threshold of 5%, and n = 6). The average numbers of detected differential proteins were reported. The average true integration-drive discovery rates (tIDR) and the average true integration revision rates (tIRR) were shown in parentheses. The results were based on 1,000 simulations.

| Meta Analysis | $\rho$=0.75 | $\rho$=0.50 | $\rho$=0.25 |
| --- | --- | --- | --- |
| Pearson’s test | 322.10 (8.20%, 2.63%) | 346.75 (5.26%, 2.79%) | 383.18% (2.88%, 1.97%) |
| Stouffer’s test | 355.05 (14.66%, 1.55%) | 376.34 (10.27%, 1.05%) | 400.04 (5.39%, 0.78%) |
